# Prenatal Attachment, Personality, and Depression in High-Risk Pregnancies During Pandemic Emergencies

**DOI:** 10.3390/healthcare12232359

**Published:** 2024-11-25

**Authors:** Sofia Burgio, Gaspare Cucinella, Giovanni Baglio, Simona Zaami, Robert Krysiak, Karolina Kowalcze, Valentina Billone, Giuseppe Gullo

**Affiliations:** 1 Maternal and Child Department with Pediatric Emergency Area, Villa Sofia—V. Cervello Hospital, 90128 Palermo, Italy; 2 Department of Obstetrics and Gynaecology, Villa Sofia—V. Cervello Hospital, University of Palermo, 90128 Palermo, Italy; gaspare.cucinella@unipa.it (G.C.); valentina.billone@gmail.com (V.B.); gullogiuseppe@libero.it (G.G.); 3 Research Unit, Italian National Agency for Regional Healthcare Services—AGENAS, 00187 Rome, Italy; baglio@agenas.it; 4 Department of Anatomical, Histological, Forensic, and Orthopedic Sciences, Sapienza University, 00198 Rome, Italy; simona.zaami@uniroma1.it; 5 Department of Internal Medicine and Clinical Pharmacology, Medical University of Silesia, Medykow 18, 40-752 Katowice, Poland; rkrysiak@sum.edu.pl; 6 Department of Pediatrics in Bytom, Faculty of Health Sciences in Katowice, Medical University of Silesia, Stefana Batorego 15, 41-902 Bytom, Poland; kkowalcze@sum.edu.pl

**Keywords:** prenatal attachment, personality traits, depression, high-risk pregnancies, COVID-19

## Abstract

**Background:** The observational study investigates how personality factors influence depression, prenatal attachment, and fear of COVID-19 in women with high-risk pregnancies. **Methods:** Women experiencing a high-risk pregnancy between the 20th and 24th weeks of gestation (N = 84) were selected. The Personality Inventory (PI), Beck Depression Inventory (BDI), Prenatal Attachment Inventory (PAI), and Fear of COVID (FCV-19S) were used for data collection. **Results:** Agreeableness was significantly negatively correlated with fear of COVID-19 (r = −0.33, *p* = 0.002) and positively correlated with prenatal attachment (r = 0.28, *p* = 0.008). Conscientiousness was negatively correlated with prenatal attachment (r = 0.34, *p* = 0.001). Depression was positively correlated with fear of COVID-19 (r = 0.27, *p* = 0.013). Linear regressions showed that agreeableness negatively predicted fear of COVID-19 (β = −0.34, *p* = 0.002) and positively predicted prenatal attachment (β = 0.27, *p* = 0.008). Conscientiousness negatively influenced prenatal attachment (β = −0.35, *p* = 0.001). **Conclusions:** This study explores personality traits in high-risk pregnancies, a variable underexplored in this clinical population. High-risk pregnancies may lead to adverse outcomes for both mother and child.

## 1. Introduction

The present study aimed to investigate how personality traits influence depression, prenatal attachment, and COVID-19 fear in women experiencing high-risk pregnancies. The research question of this study investigates the existence of a correlational relationship between personality traits and depression, prenatal attachment, and COVID-19 fear, specifically whether personality traits have a significant effect on these variables. A high-risk pregnancy is defined as a pregnancy in which there is a higher-than-average likelihood that the woman or the baby may experience health complications either before or after delivery [1]. These complications, which may arise during pregnancy, childbirth, or in the weeks following delivery, can lead to an even more fragile clinical picture if associated with psychological variables, such as particularly rigid personality traits.

It is possible to highlight how the condition characterized by a high-risk pregnancy represents a true “risk condition” for parents [2]. This condition, leading to maladaptive outcomes, can impair their individual development as well as the development of parental competence [3,4].

Here, reference is made to a model of parental competence understood as the management of parental functions related to caregiving [5], scaffolding [6], emotional coping [7], and cognitive coping [8], all of which are enacted with a child who has specific characteristics, often shaped by atypical development. 

Beyond the organic specifics, the risk condition represents the outcome of the encounter and integration between specific external risk factors and elements of parental vulnerability [9]. 

Reference is made here to the construct of psychosocial risk [9,10], in which parental vulnerability manifests through specific perceptions, representations, and relational constructs toward oneself, the child, and the reference contexts (family, professionals…) in terms of inadequacy (low self-efficacy—“I am not capable of…”, “my child won’t make it”), feelings of despair and fear about the uncertainty of the future, or the activation of defense mechanisms (delegating or denying) that are not functional for managing the stressful situation inherent in the high-risk pregnancy condition [11,12,13].

In the literature (Table 1), there are very few studies that have investigated the personality variable in women with high-risk pregnancies [14], while studies on the presence of depression and prenatal attachment as two variables capable of determining psycho-evolutionary outcomes in the postpartum period are much more widespread [15,16,17,18,19].

Specifically, the literature in this field has predominantly addressed, with a strong psychiatric focus, personality disorders in women experiencing physiological pregnancies [20,21,22] or personality traits, according to the Big Five Theory, associated with specific conditions such as pelvic girdle pain [23]. Other studies have examined how personality traits may be correlated with dysfunctional behaviors, such as alcohol and smoking use during pregnancy [24,25,26], as well as the relationship between personality traits under the Big Five Theory and fear of childbirth [27]. A notable feature of these studies is that they all focus on physiological pregnancies, without organic complications for either the mother or fetus. Therefore, the adopted framework aims to highlight how the Big Five Theory can represent an attempt to deepen the understanding of the relationship between personality traits, attachment, depressive symptoms, and fear of COVID-19.

Indeed, having a high-risk pregnancy can lead to an increased risk of depression [28], as the sense of uncertainty and fragility regarding the health of the fetus and/or the mother can, in the long term, expose women to high levels of stress and anxiety [29], which often precede more pronounced depressive conditions. Specifically, mental representations during pregnancy become the focal point where perceived self-efficacy is enacted—that is, the ability to feel competent in carrying out a task or goal, in this case, the pregnancy [30]. A reduced perception of oneself as competent, combined with a sense of physical and organic vulnerability, can therefore foster depressive experiences.

Similarly, this has a significant impact on the prenatal attachment bond [31].

A pregnant woman experiencing higher levels of depression will face greater difficulty in forming an intrapsychic connection with her child, as she confronts the potential fragility of life—a condition that is readily experienced by those going through a high-risk pregnancy [32].

Personality, on the other hand, is a construct that has been studied little in relation to depression and prenatal attachment variables in the context of high-risk pregnancies. Far from adopting a deterministic perspective, personality represents the set of distinctive traits and characteristics that shape the way an individual thinks, feels, and behaves consistently and relatively stably over time and across different situations [33].

For this reason, given that these patterns are stable and enduring, scientific interest has not focused on whether particular personality traits could be associated with a higher likelihood of experiencing elevated levels of depression and thus a compromised—or conversely, sufficiently positive—prenatal attachment. 

This near absence of studies on high-risk pregnancies is peculiar, considering that some research highlights how studying personality during pregnancy is an important indicator for clinical intervention. This is because it not only significantly improves the identification of women at increased risk of depression but also helps to identify those with an extremely low risk of depression, who may therefore have more resources to cope with the difficulties inherent in the parental role [34].

Indeed, the most prevalent psychological risk is the risk of developing depression, as widely supported by the literature in the field [35,36,37,38,39]. 

Several systematic reviews and meta-analyses, which included cohort, case–control, cross-sectional, and intervention studies involving 540,373 pregnant or postpartum women from various countries between 2000 and 2013, have demonstrated that women with high-risk pregnancies (e.g., those affected by obesity, diabetes mellitus, hyperthension cardiac pathologies, and autoimmune diseases) have a higher likelihood of both prenatal and postpartum depression compared to women with normal pregnancies (and thus within a normal weight range) [40].

Specifically, some studies also show that the presence of depressive symptoms during the prenatal period can influence the presence of depression in the postpartum period, in that high levels of prenatal depression expose women to a higher likelihood of developing depression during the transition to parenthood [15,41]. As found in some studies, prenatal depression stabilizes and continues into the postnatal period in 50% of women.

Experiencing a high-risk pregnancy can lead to feelings of sadness and disorientation, and these feelings take on clinical significance, especially if one’s personality is oriented towards a tendency to control events and lower emotional stability [42,43,44,45].

Furthermore, the increased stress experienced in high-risk pregnancies may affect the development of prenatal attachment [46], and it is well known that the mother–infant relationship during the postpartum period is strongly correlated with prenatal attachment [47,48,49].

The onset of depressive symptoms in mothers could, therefore, negatively impact their ability to attune to their baby and adequately respond to the newborn’s needs. 

As widely acknowledged, maternal responsiveness to the child’s needs encompasses behavioral, communicative, and emotional aspects, representing the mother’s ability to share both the child’s positive and negative emotions. The parent’s capacity to attune to their child’s emotions becomes an indicator of the type of attachment the child will develop [50,51] and, as a result, also serves as an indicator of adequate parenting competence.

### The Big Five Theory

Within the extensive range of personality theories in the literature, the Big Five Theory has emerged as particularly significant. This theoretical framework, rather than focusing solely on personality traits from a strictly psychopathological perspective, conceptualizes personality along a continuum, where each trait may exhibit both functional and dysfunctional dimensions of personality structure. The application of this framework to the investigation of the variables studied in high-risk pregnancies is therefore considered particularly innovative.

According to the Big Five theory, Costa and McCrae [52] propose a taxonomy of personality traits based on five broad factors: extraversion, neuroticism, agreeableness, conscientiousness, and openness to experience. 

Extraversion is a central personality trait in social behavior. Sociable, outgoing, and spontaneous individuals are more likely to form friendships and romantic relationships than introverts [53]. It has also been shown that sociability increases the likelihood of becoming parents [54]. Conversely, shyness seems to delay marriage and parenthood, particularly among men [55]. Since prosocial behaviors expand social support networks, extraversion may serve as a protective factor against high levels of depression for women experiencing high-risk pregnancies [56].

Neuroticism reflects a general tendency to experience negative emotions, such as anxiety, and to become easily distressed. It has been associated with difficulties in social relationships, such as lower quality and interpersonal negativity in marriage [57,58].

Some studies highlight that dysfunctional attachment, particularly with an anxious orientation, predicts traits with high levels of neuroticism; additionally, both anxious attachment and neuroticism mediate the relationship between childhood maltreatment and depression in adulthood [59]. Furthermore, research indicates that neuroticism shares a genetic basis with psychiatric disorders, such as major depressive disorder [60].

Neuroticism has also been studied in relation to the presence of depressive symptoms, demonstrating how this trait influences prenatal depression [61]. Understanding these data implies reflecting on the risk of postpartum depression for women experiencing depression during pregnancy, with potential psycho-evolutionary consequences on the mother–child bond [15]. Moreover, the few studies examining the relationship between maternal personality and prenatal attachment highlight that low levels of neuroticism are positively associated with prenatal attachment and the quality of the couple’s relationship [62].

Individuals high in agreeableness tend to be empathetic, caring, and cooperative, and higher levels of agreeableness are generally associated with a greater inclination toward parenthood. People with high affiliative capacity perceive parenthood more positively [63] and show less ambivalence toward parenting decisions [64], suggesting that decision-making skills and the ability to cope with developmental crises, such as those encountered in a high-risk pregnancy, are more accessible for highly agreeable individuals. Similarly, studies on women with physiological pregnancies show that high levels of agreeableness are associated with positive evaluations of pregnancy and greater effectiveness in problem solving due to functional coping strategies [45].

Conscientiousness assesses goal orientation, perseverance, and self-discipline. Studies indicate that people with physical disorders such as diabetes or hypertension, as well as mental disorders such as major depression, tend to have lower levels of conscientiousness than those without these conditions [65]. In the population with physiological pregnancies, some studies indicate that women with traits of higher conscientiousness and extraversion report fewer depressive symptoms and less stress during the peripartum period, and that in the postpartum phase, women with higher extraversion levels are less affected by the increase in depressive symptoms [66,67]. Studies on the relationship between conscientiousness and attachment, particularly from the prenatal to postnatal phases, are almost entirely absent.

Finally, openness reflects flexibility in social attitudes and worldviews, as well as cognitive and aesthetic sensitivity to stimuli. The literature presents a varied picture of the relationship between openness, depression, and prenatal attachment. On the one hand, openness, favoring curiosity and appreciation of diverse experiences, has been associated with specific mental health outcomes, including depression [68]; on the other, generally, higher levels of openness may correlate with a reduced risk of depression, as these individuals adopt reflective coping strategies that can reduce stress [69]. However, some studies suggest that high openness may also expose individuals to intense emotional experiences that, in some cases, could worsen depressive symptoms, depending on other personality traits and environmental factors [70].

A final reflection is warranted on the negative impact the COVID-19 pandemic may have had on pregnant women, causing fear and stress, subsequently influencing childbirth fear and the postpartum period. The risky situation raises questions not only about the type of high-risk pregnancy experienced by the parental couple, the specific risk conditions for the mother and/or fetus, and the possible distance of other family members, but also about the new requests from healthcare professionals to support the continuation of pregnancy and the specific family or contextual relational configurations (e.g., the presence of pre-existing problematic relationships), as well as the spaces and timing of hospital care and check-ups, which, especially during the COVID-19 pandemic, interrupted usual relational routines (familial, marital, parental…) [71,72,73,74]. 

## 2. Materials and Methods

The research design focused on an observational perspective, as the sample examined represents the group of participants at time 0 of a longitudinal study, prior to receiving any treatment. Participants were therefore involved in a research path that included multiple directions; specifically, this work aimed to focus on only a subset of the study variables, which are more thoroughly detailed in another publication (refer to the study [19]).

Given the nature of the variables, an observational study design was selected, as it allowed for the observation of relationships among the study variables at time 0, without the influence of any subsequent psychological intervention that might have altered the measured parameters.

The observational study aimed to investigate the relationship between personality factors (extraversion, agreeableness, conscientiousness, openness, neuroticism) and the variables: depression, prenatal attachment, and fear of COVID-19 in women experiencing a high-risk pregnancy. The study was conducted in accordance with the ethical principles outlined in the Declaration of Helsinki and was approved by the Palermo 2 Ethics Committee (registry number 60AOR2020 PROT. PANPEOT dated 3 December 2020).

### 2.1. Participants

Women experiencing a high-risk pregnancy between the 20th and 24th weeks of gestation (N = 84) were selected, all of whom were receiving care at a high-risk pregnancy clinic in a Sicilian hospital. The inclusion criteria were having received a diagnosis of high-risk pregnancy and understanding the Italian language for the administration of psychometric tests. 

The sample was recruited during the first gynecological visit, during which the pregnant woman was invited to participate in the research process, and informed consent for the study was provided and read. Participation was voluntary and anonymous, and the administration of the psychometric instruments took approximately 20 min. 

Below is Table 2, which summarizes the population selection process and provides the rationale for choosing the specific questionnaires used in the study.

### 2.2. Procedure and Tools

The research process involved the use of the following measures (see Table 2):

- A custom-made socio-demographic form, in which data related to sex, age, nationality, relationship status, educational level, type of occupation, presence of medical conditions, fetal issues, and any threats of miscarriage were collected.
- The Personality Inventory (PI; [75]), a self-report questionnaire consisting of 20 items designed to assess personality factors according to the Big Five model [52].
  The questionnaire includes five subscales that assess the following personality factors: extraversion, defined as a tendency toward sociability, assertiveness, positive emotionality, and excitement-seeking; conscientiousness, defined as a sense of duty and self-discipline; openness to experience, defined as intellectual curiosity; agreeableness, defined as trust in others and cooperation; and neuroticism, defined as a tendency toward emotional instability. Each item was rated on a 5-point Likert scale ranging from 1 = strongly disagree to 5 = strongly agree. Examples of items include the following: “I am rather emotionally stable” or “I spend a lot of time just having fun”. Regarding psychometric properties, the instrument shows good internal consistency (α > 0.70).
- The Beck Depression Inventory II (BDI; [76]), a self-report questionnaire consisting of 21 items, is aimed at measuring cognitive, motivational, affective, and behavioral symptoms of depression. Each item is scored from 0 to 3, with higher BDI scores indicating higher levels of depression. Examples of items include the following: “I feel sad most of the time”, “I feel sad all the time”, or “I am so sad or unhappy that I can’t stand it”. Regarding psychometric properties, the instrument shows good internal consistency (α > 0.80).
- The Fear of COVID (FCV-19S; [77]), a seven-item scale that assesses fear of COVID-19. The seven items are rated on a 5-point Likert scale from 1 (strongly disagree) to 5 (strongly agree), with scores ranging from 7 to 35. Higher scores indicate greater fear of COVID-19. Examples of items include the following: “I am very afraid of COVID-19” or “I cannot sleep because I am worried about contracting (or having) COVID-19”. Regarding psychometric properties, the FCV-19S also shows good internal consistency (α > 0.80).
- The Prenatal Attachment Inventory (PAI; [78]) is a self-report questionnaire that assesses maternal–fetal attachment as a single dimension. Examples of items include the following: “I wonder what the baby is like right now” or “I imagine calling the baby by name”. Regarding psychometric properties, the instrument shows good internal consistency (α > 0.70).

### 2.3. Statistical Analysis

For data analysis, several methodological steps were undertaken to examine and analyze the relationships among the variables of interest, utilizing various statistical techniques to gain a clearer and more detailed understanding of data behavior.

#### 2.3.1. Preliminary Analyses (Means, Standard Deviations, and Percentages)

Socio-demographic variables (see Table 3): Before examining the main study variables, descriptive analyses were performed for the socio-demographic variables (e.g., age, marital status, educational level). These analyses included calculating means (for continuous variables like age), standard deviations (to assess data variability relative to the mean), and percentages (for categorical variables like gender and marital status) to obtain a profile of the sample.

#### 2.3.2. Preliminary Analyses for Study Variables

For the main study variables (personality factors, depression, prenatal attachment, and fear of COVID-19), means and standard deviations were calculated to provide a general description of individual responses. Skewness and kurtosis were then examined:

- Skewness: This measure assessed the symmetry of the data distribution. A skewness value near zero indicates a symmetric distribution, while negative or positive values indicate a left or right skew, respectively.
- Kurtosis: This measure assessed the “peakedness” of the distribution. A high kurtosis value indicates a distribution with heavy tails (more extreme values), while a value near zero indicates a normal-tailed distribution. These statistics are helpful for understanding the nature of data distributions and whether they meet the assumptions for subsequent statistical tests.

#### 2.3.3. Pearson Correlations

Pearson correlations were calculated to examine linear relationships among the continuous variables in the study (personality factors, depression, prenatal attachment, and fear of COVID-19). This analysis provided insights into the strength and direction of associations between variables. A correlation value near +1 or −1 indicates a strong positive or negative relationship, respectively, while values near 0 indicate a weak or no relationship.

#### 2.3.4. Regression Models

To explore how personality factors influence the dependent variables (depression, prenatal attachment, and fear of COVID-19) more deeply, multiple linear regression models were conducted. Specifically:

- Independent variable: Personality factors (such as extraversion, conscientiousness, openness, agreeableness, and neuroticism) were used as predictor variables.
- Dependent variables: Depression, prenatal attachment, and fear of COVID-19 served as the dependent variables.

Linear regression was employed to determine whether personality factors (independent variables) could explain a significant portion of variability in the dependent variables (depression, prenatal attachment, and fear of COVID-19). Regression coefficients provided information on the magnitude and direction of the effect of personality factors on each dependent variable.

## 3. Results

The sample consists of 84 women, with a mean age of approximately 29.88 years and a standard deviation of 6.06, indicating some variability in participants’ ages. 

The vast majority of the sample was of Italian nationality (97.5%), while only 2.4% were foreign nationals.

Regarding relationship status, 60.7% of the women were married, 38.1% were cohabiting, and 1.2% were single. Concerning the number of children beyond the current pregnancy, 28.6% had no other children, 27.4% had one child, 29.8% had two children, 7.1% had three children, and another 7.1% had more than three children.

Educational levels varied, with the majority of women having completed middle school (56%), followed by vocational school (19%) and high school (15.5%). Only a small percentage held a bachelor’s degree (3.6%) or a PhD/specialization (1.2%).

In terms of employment status, 67.9% of the women identified as housewives, 19% were employed, 6% were unemployed, 3.6% were manual workers, 2.4% were students, and 1.2% were freelancers.

The table also provides an overview of the pathologies present in the sample: 45.3% of the women had diabetes mellitus, 23.2% had obesity, 15.4% had hypertension, 12.1% had cardiac pathologies, and 4% had autoimmune diseases.

Finally, approximately 29.8% of the women reported fetal problems, while the remaining 70.2% did not. Additionally, 20.2% had faced a threat of abortion, while 17.5% did not report this risk.

The descriptive statistics of the sample are presented in Table 3.

The means and standard deviations for the variables under study are reported in Table 4. The correlation results show that agreeableness was significantly negatively correlated with fear of COVID-19 (r = −0.33, *p* = 0.002); similarly, there was a significant positive correlation between agreeableness and prenatal attachment (r = 0.28, *p* = 0.008). Conscientiousness was significantly negatively correlated with prenatal attachment (r = 0.34, *p* = 0.001). Furthermore, the data analysis shows that depression was significantly positively correlated with fear of COVID-19 (r = 0.27, *p* = 0.013). The correlation results are shown in Table 4.

The results of the linear regressions show that agreeableness negatively predicted fear of COVID-19 (β = −0.34, *p* = 0.002); the results also indicate that agreeableness positively predicted prenatal attachment (β = 0.27, *p* = 0.008). Finally, conscientiousness significantly and negatively influenced prenatal attachment (β = −0.35, *p* = 0.001).

## 4. Discussion

The research results confirmed the correlational hypotheses between certain personality traits, prenatal attachment, depression, and fear of COVID-19. 

Additionally, as the research hypotheses indicated, a significant effect of some personality traits on attachment, depression, and fear of COVID-19 was found. 

The data analysis shows that the personality trait of agreeableness was significantly and negatively correlated with fear of COVID-19, a finding supported by the literature, as this personality trait corresponds to a greater predisposition to accept the changes brought about by the COVID-19 pandemic, along with fewer dysfunctional behaviors [79,80], as well as the use of more effective coping strategies [45]. Research suggests that people with a high level of agreeableness are also less inclined to believe misinformation, including that in favor of conservative ideologies [81]. This likely contributes to a lower tendency to accept polarized or extreme viewpoints, making pregnant women more resistant to misinformation based on division or distrust, and reducing levels related to fear of COVID-19. In this sense, pregnant women with high-risk pregnancies who scored high on agreeableness appeared less susceptible to fear of COVID-19.

Another finding supported by the literature is the positive correlation between agreeableness and prenatal attachment [82,83].

Agreeableness refers to cooperativeness, empathy, and willingness to engage with others [52]. In a study on pregnant women with preterm labor contractions, those women scored lower on the agreeableness scale compared to women with a physiological pregnancy [14]. This and other studies have shown that low agreeableness is linked to concerns during pregnancy and poor psychological health in pregnant women [14,84].

Therefore, it could be argued that in the present sample, higher levels of agreeableness corresponded to better prenatal attachment, as these women appeared more open to change and more empathetic in their intimate relationship with the fetus.

In a 2020 study, Barel and colleagues [85] highlighted how adult attachment security acted as a moderating variable, altering or influencing the association between certain temperament traits (e.g., being more curious, energetic, or empathetic) and agreeableness. Thus, with secure attachment, temperament traits had a stronger and more positive effect on agreeableness, whereas in the absence of secure attachment (or in the presence of insecure attachment), this association differed, and in some cases, was weakened or even reversed. These findings provide a prospective view supporting the positive correlation between agreeableness and prenatal attachment, suggesting that experiences within the intrauterine environment have continuity and guide development into adult life.

The results also indicate a negative correlation between conscientiousness and prenatal attachment. This finding differs from other studies, which have shown that this personality trait is associated with higher levels of mother–fetus attachment [82,86,87].

The sample of high-risk pregnant women examined in this study may have been more focused on responsibilities and high standards, typical of high conscientiousness, which involves a strong focus on adhering to rigid rules and internalized expectations regarding many aspects of life, such as obligations, good behavior, and order, sometimes at the expense of their well-being and interpersonal relationships. In this sense, this personality trait in high-risk pregnant women may have led to lower prenatal attachment.

Moreover, socioeconomic factors could play an important role in that the sample is primarily composed of Italian women who, based on their age and socioeconomic status, may adopt relatively rigid, conformist, and traditional lifestyles—as evidenced by the fact that more than two-thirds were housewives and 6% had defined themselves as “unemployed”. This would imply that partners/husbands are the main economic supporters of the family, relying on a single salary; the vast majority lived in married or cohabiting households, and three-quarters had a middle level of education. For such a demographic group, therefore, a “high-risk pregnancy” could carry the shadow of social stigma, potentially exacerbated by cultural factors.

A positive correlation supported by studies in the field is the one between depression and fear of COVID-19 [88,89,90]. 

Indeed, fear and depression related to COVID-19 are among the most common psychological problems for people who experienced pregnancy during the pandemic [91,92].

Several studies in the literature have examined the relationship between fear of COVID-19 and depression, and these studies have reported that they can trigger each other [93,94,95].

The linear regression analyses indicate that agreeableness negatively predicted fear of COVID-19. Some studies suggest that agreeableness is a good predictor of behavioral changes necessary to prevent COVID-19 infection [96]; in this sense, pregnant women with high levels of agreeableness were less concerned about infection because, as the literature shows, they more easily adopted behaviors congruent with preventing infection.

Having high levels of agreeableness in high-risk pregnant women positively predicts prenatal attachment, as indicated by the linear regressions. Other studies have also shown this significant relationship [85], supporting the notion that identifying personality traits is important in the assessment process with high-risk pregnant women, as this could potentially predict both prenatal and postnatal attachment.

Lastly, the regression analyses indicate that high levels of conscientiousness negatively predict prenatal attachment. As previously noted, this contrasts with studies in the field that support the idea that this personality trait is a predictor of good mother–fetus attachment [87]. These conflicting results further underscore the need for more in-depth studies on personality in pregnancy, especially in high-risk pregnancies, in order to gain scientifically supported knowledge.

## 5. Conclusions

The proposed study focuses on the personality traits of women with high-risk pregnancies, a variable that is underrepresented in the literature concerning this specific clinical population. High-risk pregnancies are extremely delicate conditions that can lead to maladaptive developmental outcomes for both the mother and the child [97]. Gaining deeper knowledge in this area, while considering all specific psycho-developmental outcomes, is the desired direction for future research. 

This study also encourages further reflection regarding future theoretical advancements: personality traits are important indicators to consider, particularly when thoroughly investigated in relation to other variables, such as prenatal attachment and depressive symptoms, in pandemic emergency contexts. Women with high levels of agreeableness who experienced stronger prenatal attachment highlight potential resources to build upon. Similarly, high levels of conscientiousness suggest the possibility of a risk factor for the development of the attachment bond. Therefore, future clinical interventions should incorporate these significant considerations, both from a theoretical perspective and for practical application in clinical settings.

### Limitations of the Study and Future Clinical Implication

One limitation of this study is the small number of participants and the absence of socio-demographic variables in the analysis of the relationships between the study’s variables. Factors such as age, education level, relationship status, and number of children were not considered. These variables will be taken into account in future studies.

The clinical implications of the research focus on the importance that the study of personality can have in managing potential psychological difficulties during pregnancy and in the postnatal period, especially when the historical context forces a condition of pandemic emergency. Working through the study of personality factors means supporting the patient in developing a functional attitude toward processing the situation, which can improve not only her emotional well-being but also the attachment bond formation process. From this perspective, promoting acceptance of reality, stimulating focus on short-term goals, and counteracting irrational thoughts can provide beneficial support. The challenge lies in finding an optimal balance between accepting the present medical risk and counteracting a sense of despair that could hinder the attachment bond or lead to maladaptive patterns of interaction with the newborn.

## Figures and Tables

**Table 1:** Strengths and weaknesses of the literature.

Variables Investigated	Strengths of the Literature	Weaknesses of the Literature
Psychosocial risk and high-risk pregnancy	Defines the challenges faced by parents related to high-risk pregnancy as a vulnerability factor for the development of parenting competence and individual adjustment.	Limited studies on the impact of psychosocial risk on parenting during high-risk pregnancies; limited exploration of specific psychological vulnerability factors in parents.
Parental competence	Explores models of parental competence, such as caregiving, scaffolding, and emotional and cognitive coping, applicable to contexts involving atypical child development.	Very few studies in the literature and insufficient examination of emotional and cognitive coping mechanisms in high-risk pregnancy conditions and their effects on the postnatal mother–child relationship.
Personality during pregnancy	Indicates that studying personality may aid in identifying women at risk of depression or those with strong psychological resources, enhancing preventive clinical interventions.	Lack of studies on how personality traits influence depression and prenatal attachment in high-risk pregnancies.
Depression and high-risk pregnancy	Numerous systematic reviews highlight an increased risk of depression in women with high-risk pregnancies compared to those with physiological pregnancies.	Greater need for longitudinal studies to examine the continuity of depression from the prenatal to the postnatal period.
Prenatal attachment and parenting	Highlights that prenatal attachment predicts the type of mother–child relationship in the postnatal period, essential for the development of adequate parenting competence.	Few studies exploring how the stress of high-risk pregnancy affects prenatal attachment and, consequently, long-term parenting competence.
Maternal responsiveness and emotional attunement	The literature supports that a mother’s ability to emotionally attune is an indicator of attachment type and postnatal parenting competence.	Insufficient research on the effect of prenatal depression and compromised attachment on child emotional development and parenting competence.

**Table 2:** Population selection process and rationale for the questionnaire.

Step	Process	Rationale
Population selection	Women experiencing a high-risk pregnancy between the 20th and 24th weeks of gestation (N = 84) receiving care at a Sicilian hospital’s high-risk pregnancy clinic.	This population was selected to specifically focus on the condition of high-risk pregnancy by examining various personality factors, the presence of depression as a condition with implications for mental health, the quality of prenatal attachment, and the presence of COVID-19-related fear in the context of a pandemic emergency.
Inclusion criteria	1. Diagnosis of high-risk pregnancy. 2. Fluency in Italian for comprehension and administration of psychometric tests.	Limiting the sample to high-risk cases allowed for examining how this specific pregnancy condition impacts psychological well-being. Language fluency ensured accurate responses and understanding of test items.
Recruitment method	During the wait for the gynecological appointment, patients were recruited to participate in a longitudinal study. The psychometric instruments were administered while waiting for the gynecological appointment. Specifically, the data presented here refer exclusively to time-phase 0 of the study, when no treatment had yet been implemented that would later distinguish the experimental group from the control group. Therefore, the data refer to the entire sample of women between the 20th and 24th weeks of pregnancy present in the waiting room.	This setting provided a convenient and controlled environment for explaining the study, ensuring informed consent, and maintaining confidentiality and voluntary participation.
**Questionnaire**	**Purpose**	**Justification for Use**
Personality Inventory (PI)	To assess personality factors (extraversion, conscientiousness, openness, agreeableness, neuroticism) according to the Big Five model.	The Big Five model is a widely recognized theoretical framework in psychological research; specifically, this tool allows for a comprehensive analysis of personality traits in a quick and non-demanding way, as it consists of only a few items (20) and can be easily administered during a medical waiting time.
Beck Depression Inventory II (BDI-II)	To measure depression symptoms, including cognitive, motivational, affective, and behavioral dimensions.	The BDI-II is a widely used tool for assessing depression severity. Since depression is a common risk in high-risk pregnancies, this instrument was chosen for its reliability and sensitivity to the target population
Fear of COVID (FCV-19S)	To measure the level of fear related to COVID-19.	Given the recent pandemic context, it was essential to assess fears related to COVID-19, as these may be correlated with specific personality traits. Furthermore, the test consists of only 7 items, making it easy to administer as it requires an extremely short time to complete.
Prenatal Attachment Inventory (PAI)	To evaluate maternal-fetal attachment.	The PAI is a validated measure of prenatal attachment, which is critical in understanding the psychological bond between mother and fetus in high-risk pregnancy contexts.

**Table 3:** Descriptive statistics of the sample (N = 84).

Variables	Women (N = 84)	
	Mean	SD
Age	29.88	6.06
Nationality		
Italian	97.5%	
Foreign	2.4%	
Couple’s condition		
Married	60.7%	
Cohabiting	38.1%	
Single	1.2%	
Number of children beyond pregnancy		
0	28.6%	
1	27.4%	
2	29.8%	
3	7.1%	
>3	7.1%	
Level of education:		
Primary school	4.8%	
Middle school	56%	
Professional school	19%	
High school	15.5%	
Bachelor’s degree	3.6%	
PhD/specialization	1.2%	
Job condition:		
Housewife	67.9%	
Student	2.4%	
Worker	3.6%	
Employee	19%	
Freelancer	1.2%	
Unemployed	6%	
Pathologies		
Diabetes mellitus	45.3%	
Obesity	23.2%	
Hypertension	15.4%	
Cardiac pathologies	12.1%	
Autoimmune diseases	4%	
Fetal problems		
Yes	29.8%	
No	70.2%	
Threat of abortion		
Yes	20.2%	
No	17.5%	

**Table 4:** Correlations between personality factors, depression (BDI), prenatal attachment (PAI), and fear of COVID-19 (FoC) in women with high-risk pregnancies.

Variables	1	2	3	4	5	6	7	8
1. Neuroticism	-							
2. Conscientiousness	−0.001	-						
3. Openness	0.099	0.212	-					
4. Extroversion	−0.018	0.043	0.169	-				
5. Agreeableness	0.101	−0.064	0.003	−0.045	-			
6. BDI	0.161	0.077	0.003	−0.172	−0.081	-		
7. PAI	0.008	−0.342 **	0.040	0.132	0.287 **	−0.001	-	
8. FCV-19S	0.090	0.076	0.041	−0.042	−0.336 **	0.270 **	−0.079	-
M	9.96	14.1	12.1	12.5	13.5	6.63	63.5	12.9
SD	3.19	2.56	2.46	2.16	2.63	5.64	10.8	6.76
Skewness	0.04	0.024	0.34	−0.10	0.09	1.29	−0.20	1.39
Kurtosis	0.18	−0.28	0.64	1.01	−0.26	1.51	−1.07	1.57

* *p* < 0.05; ** *p* < 0.01.

## Data Availability

The data presented in this study are available on request from the corresponding author.
